# Multi-modal imaging of high-risk ductal carcinoma in situ of the breast using C2Am: a targeted cell death imaging agent

**DOI:** 10.1186/s13058-021-01404-z

**Published:** 2021-02-17

**Authors:** Zoltan Szucs, James Joseph, Tim J. Larkin, Bangwen Xie, Sarah E. Bohndiek, Kevin M. Brindle, André A. Neves

**Affiliations:** 1 Cancer Research UK Cambridge Institute, Li Ka Shing Centre, University of Cambridge, Robinson Way, Cambridge, CB2 0RE UK; 2 Department of Physics, University of Cambridge, Cambridge, UK; 3 Present address: University of Dundee, School of Science and Engineering, Dundee, UK; 4 Department of Biochemistry, University of Cambridge, Cambridge, UK

**Keywords:** DCIS, Multi-modal imaging, Optoacoustic, Necrosis, Early detection

## Abstract

**Background:**

Ductal carcinoma in situ (DCIS) is a non-invasive form of early breast cancer, with a poorly understood natural history of invasive transformation. Necrosis is a well-recognized adverse prognostic feature of DCIS, and non-invasive detection of its presence and spatial extent could provide information not obtainable by biopsy. We describe here imaging of the distribution and extent of *comedo*-type necrosis in a model of human DCIS using C2Am, an imaging agent that binds to the phosphatidylserine exposed by necrotic cells.

**Methods:**

We used an established xenograft model of human DCIS that mimics the histopathological features of the disease. Planar near-infrared and optoacoustic imaging, using fluorescently labeled C2Am, were used to image non-invasively the presence and extent of lesion necrosis.

**Results:**

C2Am showed specific and sensitive binding to necrotic areas in DCIS tissue, detectable both in vivo and ex vivo. The imaging signal generated in vivo using near-infrared (NIR) fluorescence imaging was up to 6-fold higher in DCIS lesions than in surrounding fat pad or skin tissue. There was a correlation between the C2Am NIR fluorescence (Pearson *R* = 0.783, *P* = 0.0125) and optoacoustic signals (*R* > 0.875, *P* < 0.022) in the DCIS lesions in vivo and the corresponding levels of cell death detected histologically.

**Conclusions:**

C2Am is a targeted multi-modal imaging agent that could complement current anatomical imaging methods for detecting DCIS. Imaging the presence and spatial extent of necrosis may give better prognostic information than that obtained by biopsy alone.

**Supplementary Information:**

The online version contains supplementary material available at 10.1186/s13058-021-01404-z.

## Introduction

Breast cancer is the most commonly diagnosed malignancy in women in the Western world, affecting one in eight women during their lifetime, with approximately 20% of newly diagnosed cases being DCIS [1]. The introduction of mammographic screening programs in the 1980s led to an estimated 40% reduction in breast cancer-related mortality. However, diagnosis of biologically indolent breast cancer, which might not otherwise become clinically apparent during the lifetime of the patient [2], has led to an estimated overdiagnosis of 10% of cases, mainly attributed to the sevenfold increase in the detection rates of DCIS [3].

DCIS is a non-obligate precursor of invasive breast cancer, representing a pathologically heterogeneous group of neoplastic processes in situ, with differing growth rates, a range of microarchitectural and cytological patterns, and diverse malignant potential, with an unpredictable transition time to invasive cancer [3]. Surgical excision is mandatory in the clinical management of DCIS [4], and while mastectomy offers higher cure rates, due to its mutilating nature, this has been mostly replaced by breast-conserving surgery (BCS) [5]. There is a major clinical concern that the standard-of-care treatment of DCIS might be unnecessarily aggressive for patients with lesions deemed as “low risk” [6, 7].

The presence of *comedo*-type necrosis at diagnosis is associated with an increased risk of local recurrence, when BCS is used [8]. Both the presence and the extent of necrosis within DCIS lesions have been shown, in a sub-analysis of a large international randomized clinical trial, to have a significant association with the rates of both non-invasive and invasive local recurrences [9], particularly for high-grade lesions [10]. This trial showed that not only the presence, but also the anatomical extent of *comedo*-necrosis holds relevant prognostic information [9]. In a recent study, the presence of necrosis in diagnostic preoperative biopsies of over 600 women with DCIS was found to be a strong predictor of both DCIS upgrading and upstaging to invasive carcinoma in the final surgical specimens [11].

Reliable detection of *comedo*-type necrosis in biopsy specimens poses a challenge as it is based on a subjective assessment and is therefore highly variable [12]. At present, only post-surgical histological evaluation of breast resection specimens (lumpectomy or mastectomy) can reliably determine the actual extent of *comedo*-type necrosis. Pre-surgical diagnostic biopsy of suspect DCIS lesions can only inform on the presence or absence of this high-risk feature in the spatially limited biopsy specimen, thus providing potentially incomplete prognostic information.

The development of novel imaging tools that can predict prognosis in DCIS more effectively is an unmet clinical need [7]. Current imaging techniques do not provide prognostic information beyond anatomical estimates of lesion size, which often lack accuracy when delineating the extent of DCIS involvement. In the last two decades, magnetic resonance imaging (MRI) has emerged as a potential adjunct to mammography, despite the additional cost [13]. However, a meta-analysis concluded that routine use of preoperative MRI, in addition to conventional preoperative assessment of patients with newly diagnosed DCIS, was not associated with improved surgical outcomes [14–16]. Recently, the fast hybrid optical and acoustic technology of optoacoustic (OA) imaging has emerged as a promising diagnostic tool for the evaluation of breast lesions [17, 18]. To date, the reported OA trials have focused on imaging tumor hemoglobin concentration and oxygenation. While these metrics have been shown to correlate with tumor necrosis [19], they do not provide a specific readout of the process. Non-invasive imaging modalities that can reliably detect and potentially quantify and map *comedo*-type necrosis would be a useful adjunct to mammography.

C2Am is a modified version of the C2A domain of Synaptotagmin-I, in which a single-cysteine residue, introduced by site-directed mutagenesis, can be used for selective labeling of the protein with labels detectable using non-invasive imaging techniques [20]. This small protein (16 kDa) can detect, with low nanomolar affinity, the phosphatidylserine (PS) externalized to the outer leaflet of the plasma membrane, during apoptosis, and which becomes accessible on the inner leaflet of necrotic cells, following the plasma membrane disruption that accompanies necrosis [21]. C2Am can detect tumor cell death following treatment, using fluorescence, optoacoustic [22], SPECT [23], and more recently, PET [24] imaging in vivo. We show here that we can use a fluorophore-labeled derivative of C2Am for multi-modal detection of *comedo*-type necrosis with fluorescence and optoacoustic imaging in an established human-in-mouse intraductal transplantation model of DCIS, which has been shown to model accurately human high-risk DCIS [25].

## Materials and methods

### Cell culture

All chemicals were obtained from Sigma-Aldrich unless stated otherwise. MCF10ADCIS.com cells (CVCL_5552, Asterand, Inc. Detroit, MI) [26, 27] were stably transfected with firefly luciferase and RFP [28] (herein named MCF10A_DCIS_), cultured in DMEM/F12 (1:1) medium supplemented with 5% horse serum (Thermo Fisher Scientific) and 4 mM glutamine, in a 37 °C humidified incubator with an atmosphere of 5% CO_2_. The cell line, which tested negative for mycoplasma by an RNA capture ELISA-based method, was genotyped upon arrival and used within 4 to 8 passages from the original stocks. An automated cell viability analyzer (Vi-Cell™, Beckman Coulter) was used to monitor cell number and viability. Where required, cell death was induced by incubating cells with 10 μM doxorubicin or 100 μM etoposide (Teva, Ltd.) for 24 h at 37 °C.

### C2Am and iC2Am expression and labeling

For fluorescence and optoacoustic imaging, C2Am and a site-directed mutant of the protein that no longer binds to PS (iC2Am) [20] were expressed in *E. coli* BL21, previously transformed with pGEX-2T vector containing either GST-C2Am or GST-iC2Am [20], purified and labeled using either DyLight™-650-maleimide (Thermo Fisher Scientific, *E*_*x*_/*E*_*m*_ = 654/673 nm) for iC2Am or AlexaFluor™-750-C5-maleimide (Life Sciences, *E*_*x*_/*E*_*m*_ = 750/775 nm) for C2Am. The bioconjugation protocol has been described previously [20].

### DCIS intraductal murine model

Sub-confluent MCF10A_DCIS_ cells (> 95% viability) were washed and re-suspended at 5 × 10^3^ cells/mL, in chilled medium containing 65% DMEM/F12 (1:1), 5% horse serum, and 20% growth factor-reduced Matrigel (BD Biosciences). The cell suspension was delivered intraductally to recipient 8–12-week-old female NOD/SCID-IL2R_gamma_ mice (Jackson Laboratories), under isoflurane (1–3%) anesthesia, using a method described previously [25]. All animal experiments were performed in compliance with a project license issued under the Animals (Scientific Procedures) Act of 1986 and were designed with reference to the UK Co-ordinating Committee on Cancer Research guidelines for the welfare of animals in experimental neoplasia [29]. Protocols were approved by the Cancer Research UK Cambridge Institute Animal Welfare and Ethical Review Body.

### Planar epifluorescence (FLI) and bioluminescence (BLI) imaging

C2Am and iC2Am were administered as an equimolar mixture of 0.10 μmol/kg for each of the two probes (10 mL/kg, i.v.). Imaging in vivo and ex vivo was performed using an IVIS200™ system (Perkin Elmer). BLI was conducted 5–10 min following administration of D-luciferin (Perkin Elmer, 150 mg/kg, i.p. in phosphate-buffered saline). FLI and BLI were performed under 2–3% isoflurane gas anesthesia mixed with 100% oxygen. Body temperature was maintained at 37 °C. The in-plane resolution was 100 μm.

### Optoacoustic imaging

C2Am (C2Am-AF750) and iC2Am (iC2Am-DyL650) were administered as an equimolar mixture of 0.20 μmol/kg (10 mL/kg, i.v.). Optoacoustic (OA) imaging in vivo was performed using a multispectral optoacoustic tomography (MSOT™) inVision 256-TF small animal imaging system (iThera Medical) [30]. Briefly, a tunable optical parametric oscillator (OPO) pumped by a Nd:YAG laser provided excitation pulses with a pulse width < 7 ns at wavelengths ranging from 660 to 1300 nm at a repetition rate of 10 Hz, wavelength tuning speed of 10 ms, and peak pulse energy of 90 mJ at 720 nm. Ten arms of a fiber bundle provided uniform illumination of a ring-shaped light strip of approximately 8 mm width, with radiant exposure of < 20 mJ/cm^2^ (maximum permissible exposure) on the sample surface. For ultrasound detection, 256 toroidally focused ultrasound transducers, with a center frequency of 5 MHz (60% bandwidth) and organized in a concave array with 270° angular coverage and a radius of curvature of 4 cm, were used [31]. Anesthetized animals were wrapped in a thin polyethylene membrane and placed in the animal holder. The animal holder was held within the imaging chamber at a constant temperature (36 °C), maintained by heating degassed D_2_O. The mice were allowed to temperature stabilize for 12 min within the imaging chamber before any scans were performed. The respiratory rate of the animals was maintained (70–80 b.p.m.) by modulating the isoflurane concentration (1.5–2.5% isoflurane concentration). OA data were acquired from multiple cross-sectional slices with 10 frame averages. The OA responses of iC2Am and C2Am were evaluated using phantoms (Supplementary Figure S2).

### Immunohistochemistry and tissue fluorescence imaging ex vivo

Tissues were fixed for 24 h, using neutral-buffered formalin, prior to transfer into 70% ethanol. The sections (3 μm) were cut using a rotary microtome. Hematoxylin and eosin (H&E) staining was performed on a Leica ST5020/CV5030 workstation (Leica Biosystems). Cleaved-caspase-3 (CC3) and TUNEL staining were performed as described previously [32]. Unstained tissue sections were de-waxed and rehydrated and the slides mounted using Prolong Gold™ antifade reagent (Thermo Fisher Scientific), cured for 24 h at room temperature, prior to microscopic near-infrared fluorescence (NIRF) imaging at a resolution of 21 μm, using an Odyssey™ 250 flat-bed scanner (LiCor Inc.).

### Statistical analysis

Data are shown as mean ± SD, unless stated otherwise. A two-tailed Student’s *t* test was used for pairwise comparisons. ANOVA was used for multiple comparisons. Pearson’s *R* test was used to assess the significance of the correlations. *P* values of < 0.05 were considered significant. Statistical analyses were performed in GraphPad Prism™ (GraphPad Software, vs. 6.0).

## Results

**Fig. 1:**
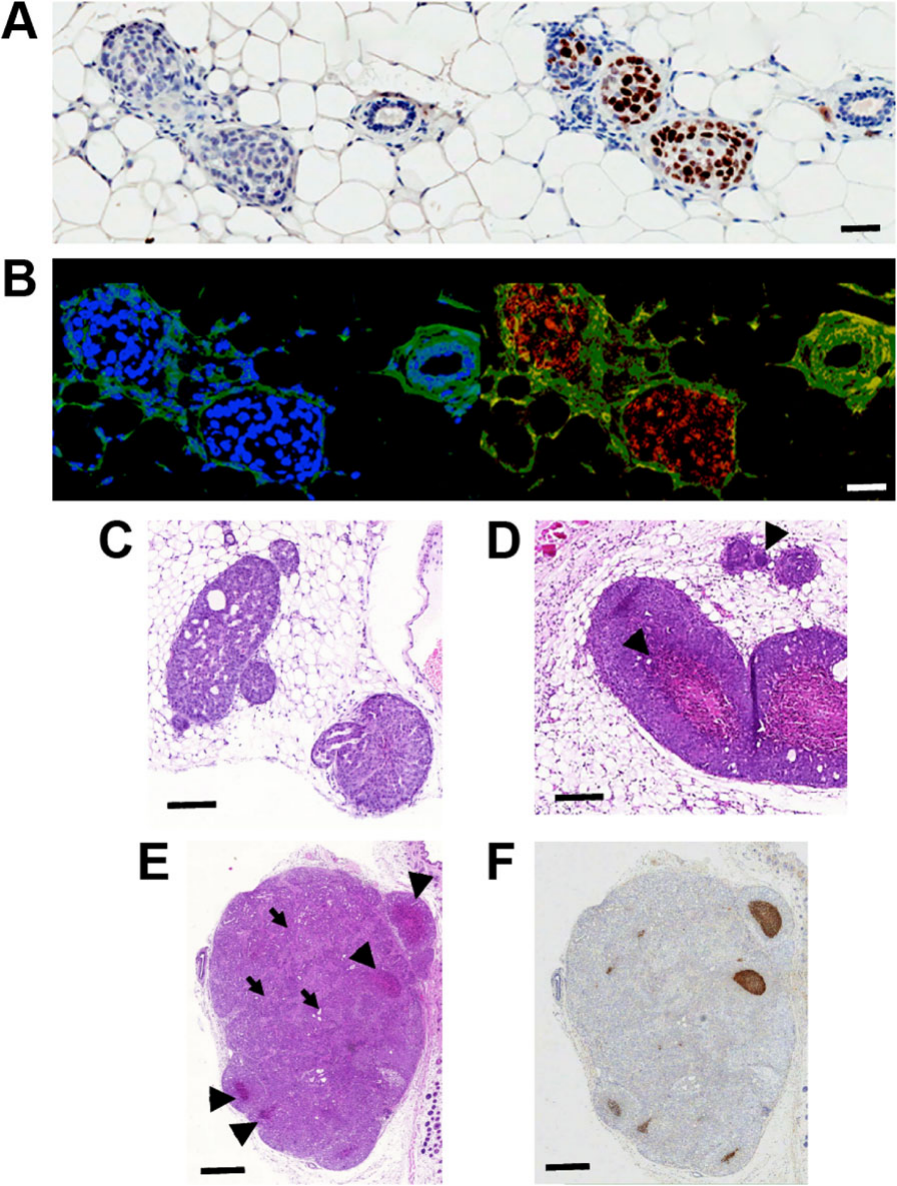
Histopathological evaluation of an intraductally transplanted model of DCIS (MCF10A_DCIS_). Two weeks post-implantation (**a, b**), solid-type intraductal growth of MCF10A_DCIS_ cells showed no staining with an antibody targeted at mouse Ki-67 but positive staining with an antibody targeted at the human protein (**a**, brown stain). DAPI staining (**a**, **b**, blue) identified both human and mouse epithelial cells whereas a FISH probe for human centromeres (**b**, red) identified cells of human origin on an autofluorescent (green) tissue background. Histopathology of DCIS lesions 8 weeks post-implantation (**c**, **d**); cribriform architecture of a MCF10A_DCIS_ xenograft (**c**); *comedo*-type necrosis in a MCF10A_DCIS_ xenograft lesion (**d**, arrowheads), with central necrotic areas. *Comedo*-necrosis type features of MCF10A_DCIS_ xenografts 8 weeks post-implantation (**e**, **f**); *comedo*-type DCIS-like (**e**, arrowheads) and invasive (**e**, arrows) structures on H&E; corresponding TUNEL staining (**f**), showing viable (gray) and necrotic (brown) regions of tissue. Scale bars = 200 μm (**a**, **b**); 350 μm (**c**, **d**); 800 μm (**e**, **f**)

The potential of MCF10A_DCIS_ cells to form DCIS-like lesions was assessed at different time points (2, 5, and 8 weeks, *n* = 10; Table 1) following intraductal implantation [33]. All lesions, manually identified at each time point, exhibited high-grade cytonuclear features, as determined by hematoxylin and eosin (H&E) staining (Fig. 1c-e). The DCIS lesion cells contained pleomorphic, irregularly spaced, and large nuclei, exhibiting marked variation in size, with irregular contours, coarse chromatin, and prominent nucleoli. The co-existence of specific architectural DCIS sub-types (solid, cribriform, *comedo*) and invasive carcinoma within each lesion was assessed. At 2 weeks following implantation, cells in DCIS lesions showed discreet intraductal growth, with no evidence of necrosis (Fig. 1a). Parallel staining, using antibodies against a mouse or human proliferation marker (Ki-67, brown), and nuclear staining (DAPI, blue) allowed the identification of human cells within the mouse mammary ducts (Fig. 1a). Staining of human centromeres (Fig. 1b, red) confirmed the extrinsic nature of the malignant cell proliferation. Lesions collected at later time points (5 and 8 weeks following implantation) showed a wider range of DCIS-specific morphological features, which resembled those of the human disease (Fig. 1c, d), including cribriform (Fig. 1c), solid, and *comedo*-type (Fig. 1d) lesions, which coexisted within the same specimen. While 5-week lesions showed incipient *comedo*-type necrosis in some (2 in 6) of the xenografts (Table 1), more mature lesions contained a larger number of *comedo*-type DCIS ducts in all of the material collected (10 out of 10, Table 1). These *comedo*-type lesions consisted of tightly packed tubular structures with central coagulative necrosis. Closer examination of H&E-stained sections (Fig. 1c) revealed a discreet basal myoepithelial layer at the periphery of the duct-like structures. Invasive areas developed in most of the older lesions (Fig. 1e, arrows; Table 1). *Comedo*-type necrosis was evident on H&E staining (Fig. 1e, arrowheads) and was identified by the presence of cell ghosts and its eosinophilic and granular nature. Additional TUNEL staining detected necrotic cell debris (Fig. 1f, brown).

**Table 1:** Morphological features of MCF10A_DCIS_ orthotopic xenograft lesions

Cohort	A	B	C
Time of tissue harvest post-implantation (weeks)	2	5	8
Number of fat pads injected (*N*)	4	6	10
Histologically confirmed DCIS/fat pads examined	2/4	6/6	10/10
Tissue architecture of DCIS lesions			
Solid	2/2	4/6	2/6
Cribriform	0/2	3/6	2/6
*Comedo*	0/2	2/6	6/6
Mixed DCIS (at least two types/specimen)	0/2	4/6	2/6
Associated invasive carcinoma	0/2	2/6	4/6

*n* = 10 mice, 18 lesions

**Fig. 2:**
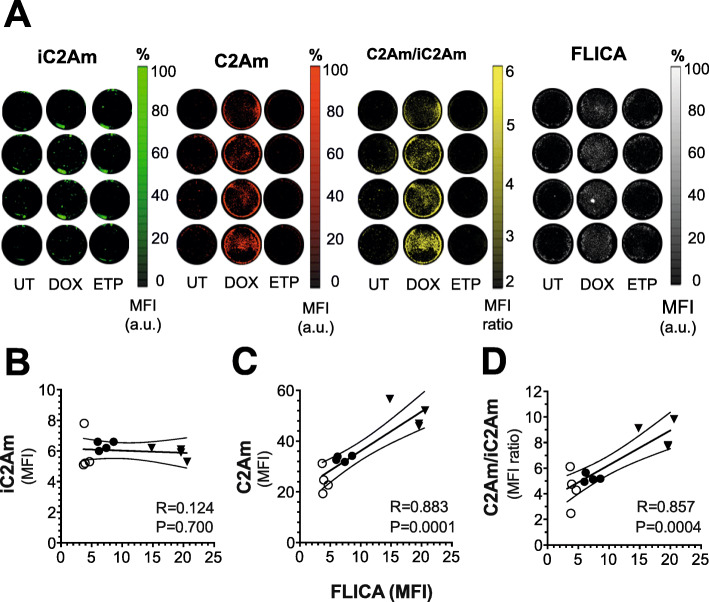
Detection of MCF10A_DCIS_ cell death in vitro using C2Am. Cells were treated with chemotherapeutic drugs (doxorubicin or etoposide) and incubated with either an equimolar mixture of C2Am|iC2Am or with a fluorescent inhibitor of effector caspases (FLICA) as a reference gold standard. Plates (**a**) were scanned at different excitation/emission wavelengths: iC2Am (650/680 nm), C2Am (780/800 nm), and FLICA (450/480 nm). Correlations of iC2Am staining (**b**), C2Am staining (**c**), and the ratio C2Am/iC2Am (**d**) with FLICA staining. UT-untreated (open circles), DOX-doxorubicin (inverted triangles), and ETP-etoposide-treated (filled circles) cells. C2Am and iC2Am were labeled with DyLigh-750 and AlexaFluor-650, respectively (see Supplementary Figure S1 and Methods). FLICA-fluorescent inhibitor of effector caspases. *n* = 4 technical replicates per experimental condition, 2 independent experiments

We assessed MCF10A_DCIS_ cell death in vitro in order to establish the capability of C2Am to detect cell death in this cell line following treatment. Cells were treated with either doxorubicin or etoposide and cell death was assessed (Fig. 2) using either an equimolar mixture of active C2Am and a site-directed mutant that does not bind PS (iC2Am) [20] or a fluorescent inhibitor of effector caspases (FLICA [34]). The combined use of C2Am and iC2Am, which were labeled with two different fluorophores, enabled a ratiometric assessment of cell death that accounted for non-specific probe retention by the cells (see Supplementary Figure S1). The level of MCF10A_DCIS_ cell death induced by doxorubicin (10 μM, 24 h, Fig. 2b, triangles) was higher than that generated by etoposide (100 μM, 24 h, Fig. 2b, filled circles), as determined by FLICA staining (Fig. 2a, right). iC2Am generated low levels of fluorescence (Fig. 2a, green), which did not correlate (*R* = 0.124, *P* = 0.7) with caspase-3 activation (Fig. 2b). C2Am, however, showed increased fluorescence with increasing levels of cell death (Fig. 2a, red), which correlated with the levels of FLICA staining (Fig. 2c; *R* = 0.883, *P* = 0.0001). The C2Am/iC2Am ratio also correlated with FLICA staining (*R* = 0.857, *P* = 0.0004), with a level of contrast that was up to 10-fold higher with C2Am than with iC2Am (Fig. 2d).

**Fig. 3:**
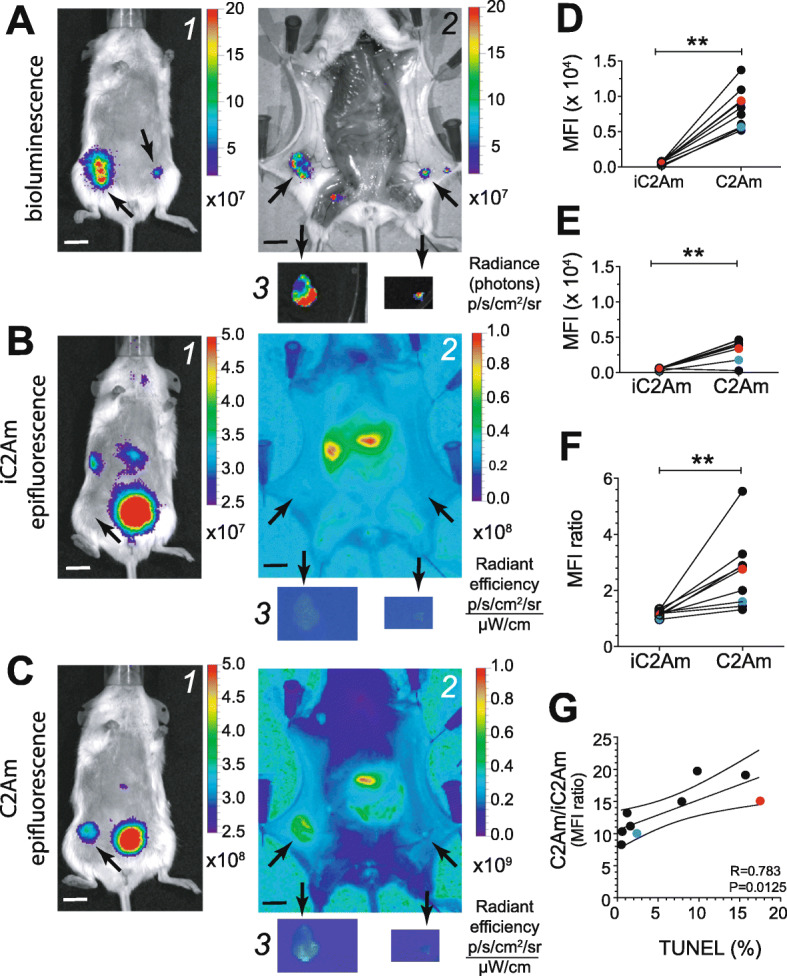
Fluorescence imaging of native cell death in the MCF10A_DCIS_ model of human DCIS. Bioluminescence (BLI, **A**) and epifluorescence images (FLI; **B**, **C**) of a representative mouse implanted intraductally with MCF10A_DCIS_ cells. Four to 8 weeks post-implantation, lesions (black arrows) were visible in vivo by BLI (**A**) and the larger lesion by FLI of C2Am in vivo (**C*1***, arrow) and both lesions ex vivo (**C*2***, arrows), but neither were visible by FLI of iC2Am in vivo (**B*1***, arrow) or easily distinguishable ex vivo (**B*2***, arrows). Lesion (**D**), fat pad (**E**), and lesion/fat pad ratios (**F**) of mean fluorescence intensity (MFI) were calculated for C2Am and iC2Am. The C2Am/iC2Am MFI ratio was also calculated for lesions and correlated with the levels of cell death, quantified by TUNEL staining of excised lesion sections (**G**). The images ex vivo (**A**–**C**: ***2***, ***3***) show the two lesions in situ in the mouse (**A*2***, **B*2***, **C*2***, arrows) and the same lesions post-resection (**A*3***, **B*3***, **C*3***) on a plate. Wilcoxon matched-pair analysis, red and blue dots (**D**–**G**) correspond to imaging data shown in (**A**–**C**), for left-side (red) and right-side (blue) lesions. **P < 0.005 (**D**–**F**). *n* = 5 mice, 9 lesions

**Fig. 4:**
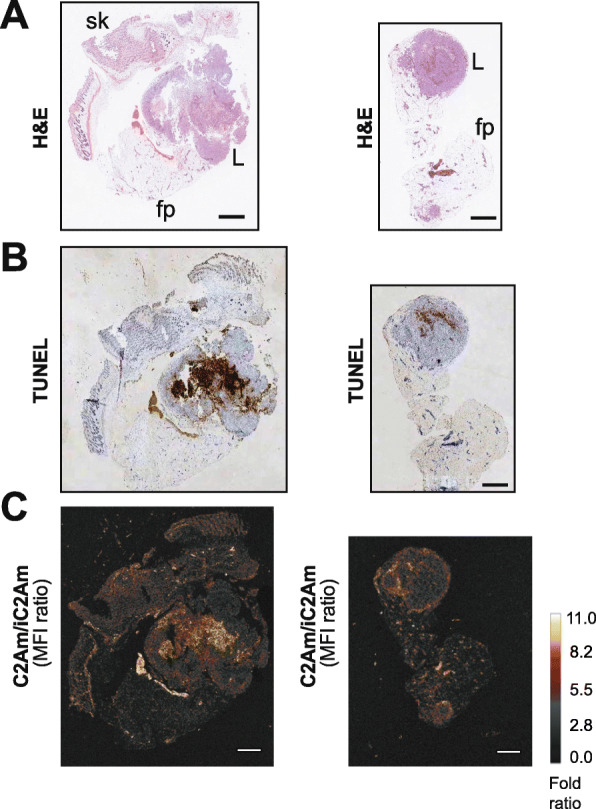
Immunohistochemistry of MCF10A_DCIS_ lesions. Representative sections from the two lesions (left and right side, respectively) resected from the mice shown in Fig. 3a-c, H&E (**a**); TUNEL (**b**), and C2Am/iC2Am ratiometric fluorescence image (**c**). L, lesion; fp, fat pad; sk, skin. Scale bars = 1.0 mm

Next, 4 to 8 weeks post-implantation of MCF10A_DCIS_ orthotopic lesions (*n* = 10), an equimolar mixture of C2Am and iC2Am was injected (i.v.) and planar epifluorescence (FLI) and bioluminescence (BLI) images were acquired at 24 h post-probe administration (Fig. 3A–C). Representative images acquired in vivo (Fig. 3A1–C1) and ex vivo (Fig. 3A2–C2 and A3–C3) are shown. The larger (∼ 7 mm) inguinal lesion detected by BLI (Fig. 3A1, left arrow), was visible in vivo using C2Am (Fig. 3C1, arrow) but not with iC2Am (Fig. 3B1, arrow), despite the fluorescence of the two probes being equivalent (Supplementary Figure S1). Both inguinal lesions (Fig. 3A1, arrows, BLI) were visible ex vivo using C2Am (Fig. 3C2, arrows) but not with iC2Am (Fig. 3B2, arrows). C2Am fluorescence-guided resection of the larger lesion (Fig. 3C1, left arrow and C3, left) showed ca. 3-fold greater (lesion/fat pad) contrast ex vivo with C2Am than with iC2Am (Fig. 3F, red symbols). Overall, lesions showed significantly higher levels (ca. 7-fold, *P* < 0.005) of fluorescence in vivo (Fig. 3D) with C2Am than with iC2Am. The relative increase in C2Am fluorescence in vivo in fat pad tissue was also significantly higher, in comparison with iC2Am, albeit the contrast was much lower than that observed for lesion tissue (Fig. 3E, ca. 1.5-fold, *P* < 0.005). The lesion-to-fat pad contrast in vivo was also ca. 3-fold higher (Fig. 3F, *P* < 0.005,), using the active C2Am probe. Moreover, the ratiometric (C2Am/iC2Am) assessment of cell death in vivo correlated (Fig. 3G, *R* = 0.783, *P* = 0.0125) with the assessment of lesion cell death ex vivo using the TUNEL staining assay. Subsequent histochemical assessment of excised lesion sections (Fig. 4) showed good co-localization of the ratiometric C2Am/iC2Am signal (Fig. 4c) with lesion cell death detected by TUNEL staining (Fig. 4b), for the two lesions present in this mouse (Fig. 3A1).

**Fig. 5:**
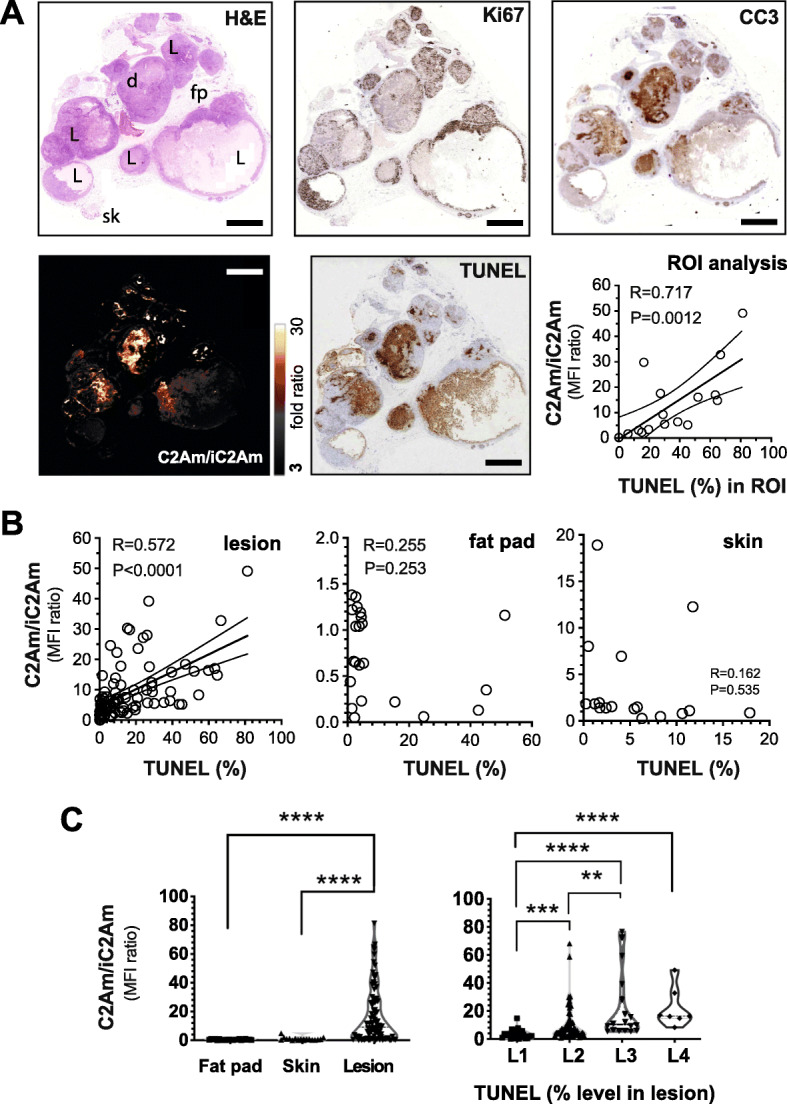
Immunohistochemistry of MCF10A_DCIS_ lesions. Serial histological sections (**a**) of one representative MCF10A_DCIS_ lesion resected 8 weeks post-intraductal cell implantation. H&E, Ki-67, CC3, TUNEL, and C2Am/iC2Am ratiometric fluorescence signals are shown. Chart (**a**), correlation of the C2Am/iC2Am ratiometric signal with the percentage of cell death, determined by TUNEL assay, for regions of interest (ROI) of the lesion shown in **a**. L, lesion; fp, fat pad; sk, skin. Correlation of C2Am/iC2Am ratiometric signal (**b**) from multiple lesions, fat pad, and skins from several mice with the corresponding levels of TUNEL staining. C2Am/iC2Am ratiometric signal (**c**) in multiple fat pad, skin, and lesions. L1: < 10% TUNEL positivity, L2: 10–25%, L3: 25–50%, L4: > 50%. **b**, **c**, *n* = 10 mice, 89 lesions, 22 fat pad, 17 skin samples. **c** Two-tailed, *t* test, unequal variance. **P < 0.005, ***P < 0.001, ****P < 0.0001. Scale bars (**a**) = 2 mm

Next, we analyzed multiple excised lesion sections from a cohort of animals (*n* = 10), 4 to 8 weeks following intraductal implantation of MCF10A_DCIS_ cells. H&E staining (Fig. 5a, top left) of representative lesions showed clear ductal lesion structures (L) embedded in fat pad tissue (fp) and surrounded by skin (sk). Within these lesions, there were areas with variable cell proliferation (Fig. 5a, Ki-67-stained). Defined, punctate areas of necrosis visible in the lesions (Fig. 5a, H&E) were also detected by cleaved caspase-3 (Fig. 5a, CC3) and TUNEL (Fig. 5a, TUNEL) staining. In regions of interest (ROI) within these lesions, the ratiometric C2Am/iC2Am signal showed a strong correlation (*R* = 0.717, *P* = 0.0012) with TUNEL staining (Fig. 5a, ROI analysis). A correlation was also found between the ratiometric C2Am/iC2Am signal and TUNEL staining for a large panel of lesions (Fig. 5b, lesion) but not for fat pad (Fig. 5b, fat pad) or skin (Fig. 5b, skin). The contrast obtained with the ratiometric C2Am/iC2Am signal (Fig. 5c, left) was also significantly greater for lesions, in comparison with surrounding fat pad (30-fold, *P* < 0.0001) and skin (15-fold, *P* < 0.0001), and increased in proportion to the levels of cell death detected by TUNEL staining in those lesions (Fig. 5c, L1–L4).

**Fig. 6:**
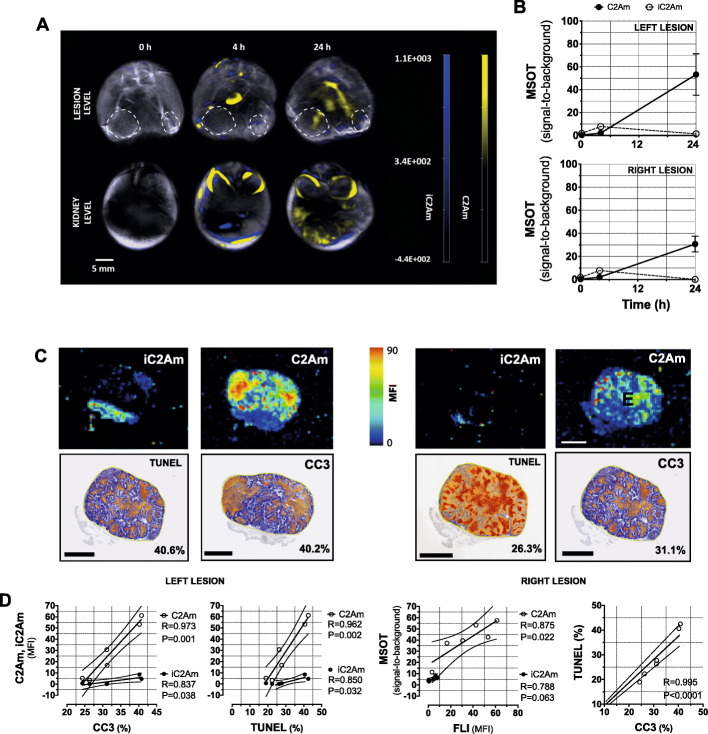
Multi-modal imaging of native cell death in the MCF10A_DCIS_ model of human DCIS. Two lesions were implanted in the lower mammary glands of three mice. Time series of axial optoacoustic images of C2Am and iC2Am, at the level of the lesion (**a**, top), and kidneys (**a**, bottom) in a representative animal. Time courses of optoacoustic (MSOT) signal in the two lesions are shown (**b**). Fluorescence imaging (FLI) ex vivo (**c**, top) of histological sections of the lesions shown in **a**. Histological sections (**c**, bottom) of lesions in **a** stained for CC3 and TUNEL; areas of staining (orange/brown) are highlighted against background signal (blue); level of cell death in the lesion is shown (lower right, %). Pearson correlation analyses (**d**) of C2Am and iC2Am optoacoustic signals with CC3 and TUNEL staining, of C2Am and iC2Am optoacoustic signals with the corresponding fluorescence signals, and of TUNEL staining with CC3 staining. Open circles (C2Am); closed circles (iC2Am). *n* = 3 mice, 6 lesions. Scale bars (**a**, **b**) = 10 mm, (**c**, **d**) = 4 mm. Dashed lines (**a**, top) indicate lesion location

Lastly, with a view to clinical translation, we conducted optoacoustic imaging of cell death in the same DCIS model using C2Am and iC2Am. Six to 8 weeks post-orthotopic implantation of MCF10A_DCIS_ cells, an equimolar mixture of C2Am and iC2Am was injected (i.v.) and optoacoustic imaging (Fig. 6a, b) and FLI (Fig. 6c) were performed in vivo and ex vivo, respectively, at 4 and 24 h post-probe administration. There were strong correlations between C2Am lesion MSOT signal and cell death markers (Fig. 6d), CC3 (Fig. 6d, *R* = 0.973, *P* = 0.001), and TUNEL (Fig. 6d, *R* = 0.962, *P* = 0.002) and also between C2Am lesion MSOT and FLI signals (Fig. 6d, *R* = 0.875, *P* = 0.022). iC2Am lesion MSOT and FLI signals (Fig. 6d) did not correlate with the cell death markers.

## Discussion

Several cell surface markers have been proposed as targets for the molecular imaging of DCIS, including IGF1-R, VEGFr, HER2, EGFR, MET, CD44v6, GLUT1, CA-XII, and mammaglobin [35]. However, to date, none of these biomarkers has been generally adopted as a predictor of DCIS recurrence [36]. Fluorescence imaging of CA-IX, a marker of hypoxia, has been suggested for better delineation of DCIS margins at surgery [37]. However, hypoxia lacks specificity as a marker of DCIS, since it can be a feature of any highly proliferative process, either benign or malignant [38], particularly in breast ducts.

Molecular markers, including hormone receptors (estrogen or progesterone) and HER-2 expression status, have been used previously as imaging biomarkers of breast cancer [39, 40]. However, these breast cancer biomarkers have not been established as prognostic features of DCIS [41], despite their clinical relevance as prognostic factors in breast cancer [42]. In contrast, necrosis is a highly specific, pathognomonic feature of DCIS, present in up to 93% of all DCIS cases [38, 43] and rarely exhibited (< 3%) by other benign or high-risk proliferative conditions of the breast [44], and which has been used as a prognostic marker of DCIS for several decades [45]. The UKCCCR/ANZ DCIS trial demonstrated that not only the presence but also the anatomical extent of DCIS-associated necrosis are adverse prognostic features of the disease, and both are strong predictors of invasive recurrence [9]. Moreover, in a 10-year follow-up study of 728 patients, the presence of confluent tumor necrosis in invasive areas of lymph-node positive breast cancer, within 2 years of diagnosis, was found to be an independent predictor for early recurrence and overall survival [46].

Owing to the significant concern of overdiagnosis and associated overtreatment, there are several ongoing clinical trials exploring treatment de-escalation in the management of DCIS [47, 48]. These trials compare active patient surveillance with the current standard of care (mandatory surgical excision) and have excluded patients with DCIS harboring adverse prognostic features, including the presence of *comedo*-necrosis at biopsy. However, reliable detection of *comedo*-type necrosis in biopsy specimens shows significant variability, even among expert pathologists [12]. Moreover, segments of DCIS containing the *comedo* sub-type can be potentially missed, since a single biopsy cannot be informative of the extent of DCIS present in the entire network of breast ducts. Approximately 10% of patients, deemed eligible as “low risk” DCIS for the ongoing COMET, LORD, and LORIS trials based on the initial diagnostic biopsy, have been estimated to belong to the “high-risk” group [9].

To our knowledge, the use of DCIS-associated necrosis as an imaging biomarker of the diseased breast has not yet been explored. Non-invasive molecular imaging of *comedo*-type necrosis could serve as an adjunct modality in the selection of patients for active surveillance. Moreover, it could also provide complementary prognostic information on the extent of aggressive, high-risk disease that cannot be obtained by biopsy.

Annexin-V (35 kDa) is a PS-binding protein that has been tested in the clinic as a cell death imaging agent. However, it showed suboptimal contrast and unfavorable pharmacokinetics [49]. We have used here C2Am, a smaller (16 kDa) cell death imaging agent, which also detects PS exposure, to detect *comedo*-type necrosis in DCIS in a well-characterized human-to-mouse xenograft model (Fig. 1). The probe was used in combination with an inactive counterpart (iC2Am) and showed high specificity for detecting cell death in vitro (Fig. 2), in vivo (Figs. 3 and 6), and ex vivo (Fig. 4). Moreover, C2Am demonstrated high sensitivity for detecting the relatively low levels of cell death present in MCF10A_DCIS_ lesions (< 5%) in vivo (Fig. 3g) and ex vivo (Fig. 4) and over a wide dynamic range (up to 20% in vivo, Fig. 3g, and up to 80% ex vivo, Fig. 5a). High levels of contrast were obtained for lesion/fat pad (30-fold) and lesion/skin (15-fold) (Fig. 5c, left), and the ratiometric C2Am/iC2Am signal was shown to correlate with the levels of cell death (Fig. 5c, right) detected by TUNEL staining.

Recent reports using optoacoustic techniques have demonstrated the capabilities of the technique for breast imaging [18]. We showed that the signal obtained from C2Am (Fig. 6a, b) using multispectral optoacoustic tomography (MSOT) correlated well with that obtained using fluorescence imaging (Fig. 6c, top). Moreover, the C2Am MSOT signal also correlated well with histological markers of cell death, CC3, and TUNEL (Fig. 6d), whereas iC2Am generated significantly less signal (*P* < 0.05, *n* = 6) in the same lesions (Fig. 6d). Beyond wide-field epi-illumination fluorescence imaging, optoacoustic methods can offer high-resolution and cross-sectional imaging at several centimeters depth [17]. C2Am-based imaging of cell death using optoacoustic imaging could bring additional prognostic value to DCIS imaging, complementing mammography and other anatomical imaging modalities, which could prove particularly beneficial for the secondary surveillance of women with an increased risk of breast cancer [50]. Optoacoustic imaging is a bed-side technique that could provide a faster and cheaper diagnostic method than MRI.

## Conclusions

Clinical translation of C2Am for imaging the presence and spatial extent of necrosis in DCIS may provide valuable prognostic information that is not obtainable from biopsy alone.

## Supplementary Information

**Additional file 1: Figure S1** Fluorescence imaging (**a**) of C2Am and iC2Am standard solutions. Excitation (dashed) and emission (filled) profiles (**b**) of iC2Am-650 (Ex/Em: 652|672 nm, red, left) and C2Am-750 (Ex/Em: 753|783 nm, brown, right). Serial dilutions (decreasing concentration, left to right) of the imaging probes (**c**, **e**) were imaged on an IVIS200™ system and the mean fluorescence intensity (in radiant efficiency) correlated with probe concentration, in the far red (**d**) and near infra-red NIR (**f**), for C2Am (open circles) and iC2Am (closed squares). iC2Am fluorescence (**d**) in the far-red (black squares) was minimal (ca. 10% of that of C2Am, open circles). C2Am fluorescence (**f**) in the NIR was undetectable. Fluorescence intensities (**g**) of C2Am-650 (in the far-red, open circles in **g**, data in 2nd row of **c**) and iC2Am-750 (in the NIR, closed squares in **g**, data in the top row of **e**) were identical at all concentrations. A 1:1 molar mixture (**h**) of C2Am|iC2Am (**c, e,** lower row) was also imaged in the far-red (open circles) and NIR (closed squares) showing equivalent readouts at all concentrations, in the range 40-2,500 nM. **Figure S2.** Single wavelength OA images of (**a**) phantoms (**b**) and in vivo tumor models are illustrated with the regions of interest (ROIs) used for the data analysis outlined. (**c**) OA spectra of iC2Am and C2Am molecules were obtained from multispectral imaging of the probes using tissue mimicking phantoms.

## Data Availability

The raw data acquired during this study and on which the results presented in this paper are based can be found at 10.17863/CAM.64395.

## Abbreviations

BLI: Bioluminescence imaging
BCS: Breast-conserving surgery
CC3: Cleaved-caspase-3 histological assay for apoptosis
COMET: Comparison of Operative to Monitoring and Endocrine Therapy clinical trial
DCIS: Ductal carcinoma in situ
FLI: Planar fluorescence imaging
H&E: Hematoxylin and eosin
LORD: LOw-Risk DCIS clinical trial
LORIS: LOw-RISk DCIS clinical trial
MCF10ADCIS: MCF10ADCIS.com breast cell line transfected with firefly luciferase and RFP
NIR: Near-infrared fluorescence imaging
OA: Optoacoustic imaging
PS: Phosphatidylserine
RFP: Red fluorescent protein
TUNEL: Terminal deoxynucleotidyl transferase dUTP nick-end labeling histological assay for necrosis
