# Correction to: Multi-modal imaging of high-risk ductal carcinoma in situ of the breast using C2Am: a targeted cell death imaging agent

**DOI:** 10.1186/s13058-021-01414-x

**Published:** 2021-03-15

**Authors:** Zoltan Szucs, James Joseph, Tim J. Larkin, Bangwen Xie, Sarah E. Bohndiek, Kevin M. Brindle, André A. Neves

**Affiliations:** 1 Cancer Research UK Cambridge Institute, Li Ka Shing Centre, University of Cambridge, Robinson Way, Cambridge, CB2 0RE UK; 2 Department of Physics, University of Cambridge, Cambridge, UK; 3 Present address: School of Science and Engineering, University of Dundee, Dundee, UK; 4 Department of Biochemistry, University of Cambridge, Cambridge, UK

**Correction to: Breast Cancer Res (2021) 23:25**

**https://doi.org/10.1186/s13058-021-01404-z**

After publication of the original article [1], the authors identified two errors in Figure 6.

The correct figure is given below.

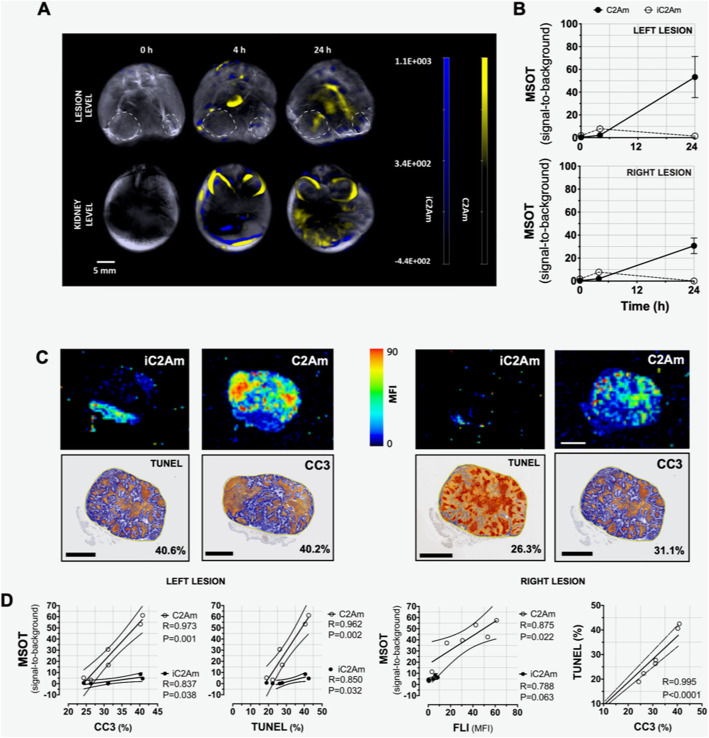



The original article has been corrected.
