# Supplementary material for: Multi-modal imaging of high-risk ductal carcinoma in situ of the breast using C2Am: a targeted cell death imaging agent
Source: Breast Cancer Res. 2021 Feb 17;23:25. doi: 10.1186/s13058-021-01404-z (PMC7891030; doi:10.1186/s13058-021-01404-z)
Supplement: Supplementary file 1 — Additional file 1: Figure S1 Fluorescence imaging (a) of C2Am and iC2Am standard solutions. Excitation (dashed) and emission (filled) profiles (b) of iC2Am-650 (Ex/Em: 652|672 nm, red, left) and C2Am-750 (Ex/Em: 753|783 nm, brown, right). Serial dilutions (decreasing concentration, left to right) of the imaging probes (c, e) were imaged on an IVIS200™ system and the mean fluorescence intensity (in radiant efficiency) correlated with probe concentration, in the far red (d) and near infra-red NIR (f), for C2Am (open circles) and iC2Am (closed squares). iC2Am fluorescence (d) in the far-red (black squares) was minimal (ca. 10% of that of C2Am, open circles). C2Am fluorescence (f) in the NIR was undetectable. Fluorescence intensities (g) of C2Am-650 (in the far-red, open circles in g, data in 2nd row of c) and iC2Am-750 (in the NIR, closed squares in g, data in the top row of e) were identical at all concentrations. A 1:1 molar mixture (h) of C2Am|iC2Am (c, e, lower row) was also imaged in the far-red (open circles) and NIR (closed squares) showing equivalent readouts at all concentrations, in the range 40-2,500 nM. Figure S2. Single wavelength OA images of (a) phantoms (b) and in vivo tumor models are illustrated with the regions of interest (ROIs) used for the data analysis outlined. (c) OA spectra of iC2Am and C2Am molecules were obtained from multispectral imaging of the probes using tissue mimicking phantoms. [file 13058_2021_1404_MOESM1_ESM.docx]

**Multi-modal imaging of high-risk ductal carcinoma *in situ* of the breast using C2Am: a targeted cell death imaging agent**

Zoltan Szucs^1^, James Joseph^1,2^, Tim Larkin^1^, Bangwen Xie^1^, Sarah E. Bohndiek^1,2^, Kevin M. Brindle^1,3^ and André A. Neves^1^

*^1^Cancer Research UK Cambridge Institute, Li Ka Shing Centre, ^2^Department of Physics, ^3^Department of Biochemistry; University of Cambridge, Cambridge, United Kingdom.*

**METHODS**

**C2Am and iC2Am chemical labeling**

Briefly, the proteins were reduced at room temperature, for 30 min, in HEPES-buffered saline (HBS buffer: 20 mM HEPES, 150 mM NaCl, pH 7.4) containing 10 mM dithiothreitol (DTT). The reduced protein was washed with ice cold HBS in a 5-kDa Vivaspin™ (Sartorius) and concentrated to 1 mg/mL, prior to the drop-wise addition of the maleimide-fluorophore (8× molar excess, in dimethyl sulfoxide, DMSO). Reaction was conducted at room temperature with stirring (200 r.p.m.) and quenched by dilution in glycine buffer (0.6 M, pH 9.2) to a concentration of 0.05 M. The final conjugates of C2Am-650 or iC2Am-750 were concentrated using Vivapsin concentrators and analyzed by ESI-MS using the method described previously (1).

**C2Am and iC2Am spectral calibrations**

For spectral calibrations**,** serial dilutions of the two conjugates were prepared in HBS buffer, in the concentration range of 0-500 nM, using black multi-well plates. An additional series was prepared using a 1:1 mixture of iC2Am-650|C2Am-750. The plates were imaged (Supplementary Fig. S1) using a Xenogen IVIS200™ system (Perkin Elmer) using far red (Ex|Em: 615-665|695-770 nm) and near infra-red (NIR) (Ex|Em: 705-780, 810-885 nm) filter sets and the resulting data analyzed using Living Image™ software (vs. 4.4, Perkin Elmer).

**DCIS intraductal murine model.**

A 30-gauge Hamilton™ syringe, 25-μL capacity, with a blunt-ended ½” needle was used to deliver the cells according to the procedure described by Behbod et al. (2). The nipple of a mammary gland (inguinal or thoracic) of the anesthetized mouse was snipped so that the needle could be directly inserted through the nipple. Ten microliters of cell suspension (containing 0.1% v/v/ trypan blue) were delivered per gland. Cells were injected into a maximum of two inguinal and one thoracic gland per mouse. The injected cell suspension could be detected visually in the ductal tree. Tumors were allowed to develop for between 4−6 weeks.

**Immunohistochemical staining of cell proliferation and death.**

Ki-67 staining was performed in the Bond-Max™ system, using rat anti-mouse (M7249, 1:500), or mouse anti-human (M7240, 1:200) antibodies (Dako). Cleaved-caspase-3 (CC3) assay was performed used a rabbit monoclonal anti-CC3 antibody (Cell Signaling Technology; 1:100 dilution) and a donkey anti-rabbit secondary biotinylated antibody (Jackson ImmunoResearch Laboratories; 1:250 dilution in Bond^TM^ diluent), on a Leica Microsystems (LM) Bond-Max™ system (Leica Biosystems), using a LM Bond Intense-R Detection Kit™, based on streptavidin-HRP (horse-radish-peroxidase) for detection of the secondary antibody (3). An avidin-biotin blocking kit (Vector Laboratories) was used. The TUNEL (TdT-mediated dUTP Nick-End Labeling) assay was used on formalin-fixed paraffin-embedded tissues and based on the DeadEnd^TM^ Colorimetric system (Promega), in conjunction with LM’s Polymer Refine Detection™ system (4). A hematoxylin nuclei counterstain was used for both the CC3 and TUNEL assays. Fluorescent inhibitor of effector caspase (FLICA™, ImmunoChemistry Technologies) cell staining followed the method described previously (1). A Zeiss Mirax™ Scan 150 (Carl Zeiss), was used with a 20x objective for digitization (at 0.369 μm/pixel resolution) of tissue sections, which were stored using a Spectrum^TM^ digital pathology information database (Aperio™). Aperio’s ImageScope™ software was used for analysis of the digitized images.

**
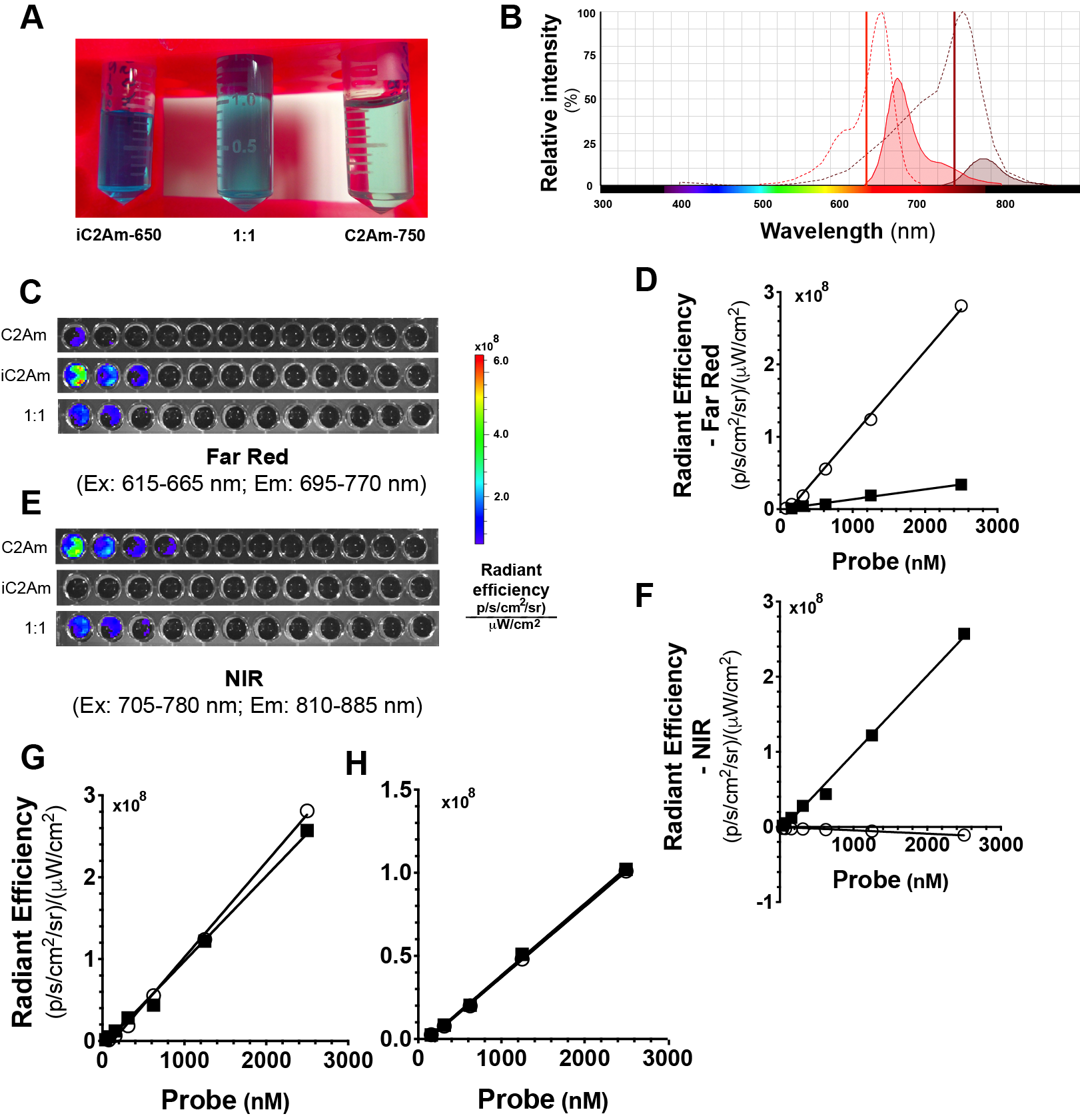
**

**Supplementary Fig. S1** Fluorescence imaging (**a**) of C2Am and iC2Am standard solutions. Excitation (dashed) and emission (filled) profiles (**b**) of iC2Am-650 (Ex/Em: 652|672 nm, red, left) and C2Am-750 (Ex/Em: 753|783 nm, brown, right). Serial dilutions (decreasing concentration, left to right) of the imaging probes (**c**, **e**) were imaged on an IVIS200™ system and the mean fluorescence intensity (in radiant efficiency) correlated with probe concentration, in the far red (**d**) and near infra-red NIR (**f**), for C2Am (open circles) and iC2Am (closed squares). iC2Am fluorescence (**d**) in the far-red (black squares) was minimal (ca. 10% of that of C2Am, open circles). C2Am fluorescence (**f**) in the NIR was undetectable. Fluorescence intensities (**g**) of C2Am-650 (in the far-red, open circles in **g**, data in 2^nd^ row of **c**) and iC2Am-750 (in the NIR, closed squares in **g**, data in the top row of **e**) were identical at all concentrations. A 1:1 molar mixture (**h**) of C2Am|iC2Am (**c, e,** lower row) was also imaged in the far-red (open circles) and NIR (closed squares) showing equivalent readouts at all concentrations, in the range 40-2,500 nM.

**Optoacoustic Image Analysis**

Optoacoustic images were reconstructed offline and analyzed using ViewMSOT software package (v3.8; iThera Medical GmbH). For in-vivo image analysis, images were reconstructed using the model-linear based reconstruction with a pixel size of 100µm x 100µm. For phantom studies were performed to estimate the OA response of the molecules (Supplementary figure S2A and 2C). Phantom images were reconstructed using the model-linear based reconstruction with a pixel size of 75µm x 75µm. Image reconstructions accounted for the spatial and temporal impulse response of the transducer, with a pass band between 50 kHz and 7 MHz. Similar speed of sound values were used for all image reconstructions within a given data set. For spectral processing linear regression based un-mixing (with pre-loaded spectra of iC2am, C2Am, HbO2 and Hb values) within the software package were used. ROI’s that cover the entire tumor area were drawn on per slice basis to obtain the OA signal from the entire tumor volume (see Supplementary figure S2B). Background ROIs were also drawn on healthy tissue region near the spine for corresponding image slices. Signal-to-background (SBR) for the volume was calculated from averaging the ratio of the mean of the signal to the mean of the background values.


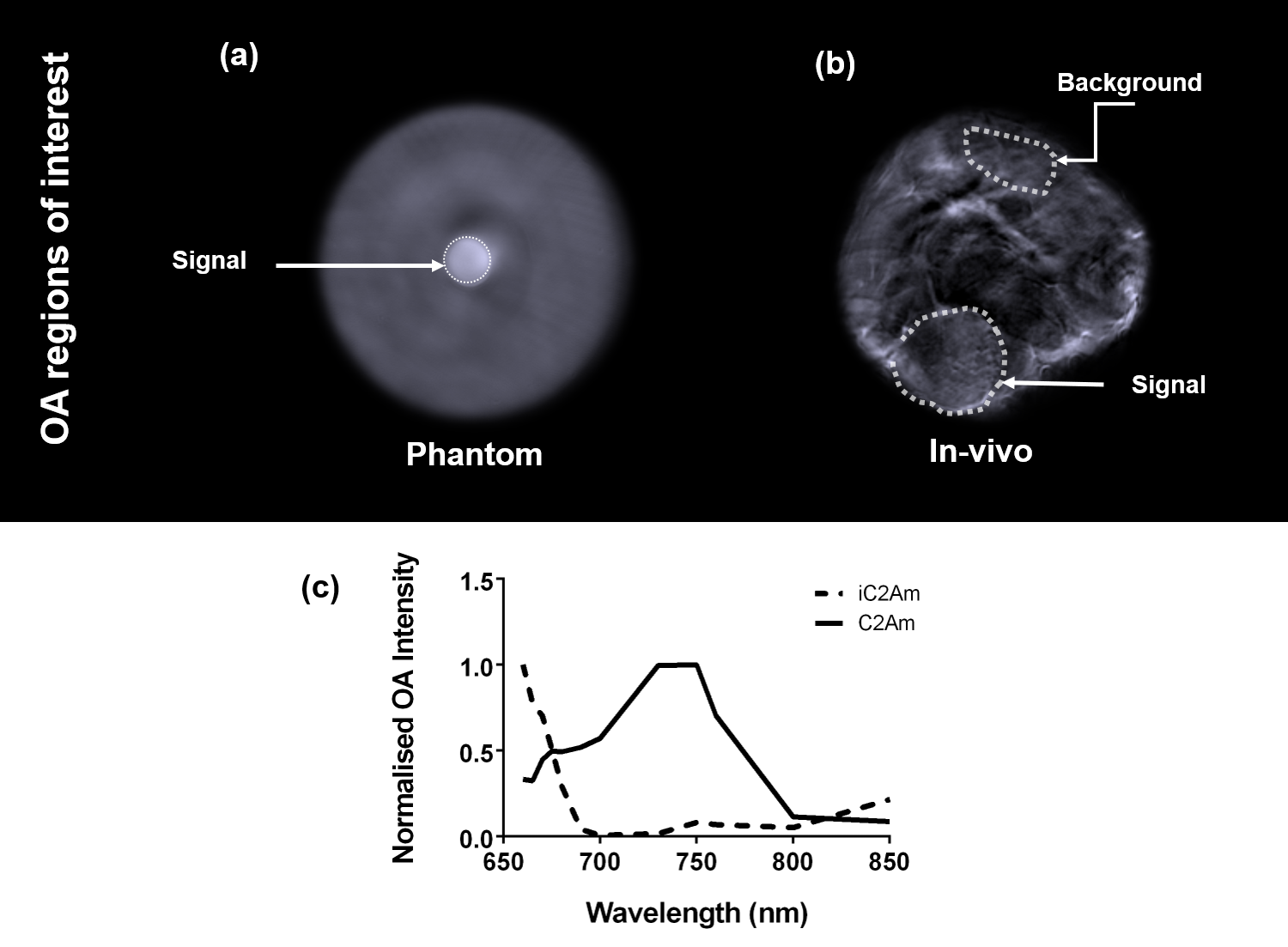


**Supplementary Fig. S2.** Single wavelength OA images of (**a**) phantoms (**b**) and in vivo tumor models are illustrated with the regions of interest (ROIs) used for the data analysis outlined. (**c**) OA spectra of iC2Am and C2Am molecules were obtained from multispectral imaging of the probes using tissue mimicking phantoms.

**References**

1. Alam IS, Neves AA, Witney TH, Boren J, Brindle KM. Comparison of the C2A domain of Synaptotagmin-I and Annexin-V as probes for detecting cell death. Bioconjug Chem. 2010;21:884-91.

2. Behbod F, Kittrell FS, LaMarca H et al. An intraductal human-in-mouse transplantation model mimics the subtypes of ductal carcinoma in situ. Breast Cancer Res. 2009;11:R66.

3. Xie B, Tomaszewski MR, Neves AA et al. Optoacoustic Detection of Early Therapy-Induced Tumor Cell Death Using a Targeted Imaging Agent. Clin Cancer Res. 2017;23:6893-903.

4. Hesketh RL, Wang J, Wright AJ et al. Magnetic Resonance Imaging Is More Sensitive Than PET for Detecting Treatment-Induced Cell Death-Dependent Changes in Glycolysis. Cancer Res. 2019;79:3557-69.
